# Initial evidence that non-clinical autistic traits are associated with lower income

**DOI:** 10.1186/s13229-017-0179-z

**Published:** 2017-11-13

**Authors:** William J. Skylark, Simon Baron-Cohen

**Affiliations:** 1 Department of Psychology, University of Cambridge, Cambridge, CB2 3EB UK; 2 Autism Research Centre, Department of Psychiatry, University of Cambridge, Cambridge, CB2 8AH UK

## Abstract

Among non-clinical samples, autistic traits correlate with a range of educational and social outcomes. However, previous work has not investigated the relationship between autistic traits and income, a key determinant of socio-economic status and well-being. In five studies (total *N* = 2491), we recruited participants without a diagnosis of autism from the general US population via an online platform and administered the short-form Autism Spectrum Quotient (AQ) as well as asked a range of demographic questions. We found a negative association between AQ and household income, which remained robust after controlling for age, gender, education, employment status, ethnicity, and socially desirable responding. The effect was primarily driven by the participant’s own income and was mainly due to the social subscale of the AQ. These results provide initial evidence that income is negatively related to autistic traits among the general population, with potential implications for a range of social, psychological, and health outcomes.

**Electronic supplementary material:**

The online version of this article (10.1186/s13229-017-0179-z) contains supplementary material, which is available to authorized users.

## Background

Autism spectrum conditions (henceforth autism) represent the upper end of a distribution of traits found in the general population [1–3]. Building on the observation that the relatives of people with autism often show the broader autism phenotype (BAP) (that is, sub-clinical levels of the atypical social, communicative, and repetitive behaviours that characterize autism [4, 5]), researchers have employed a number of self-report measures to gauge the presence of autistic traits in representative, typical samples. These include the Autism Spectrum Quotient (AQ; [1]), the Broad Autism Phenotype Questionnaire (BAPQ; [6]), the Subthreshold Autism Trait Questionnaire (SATQ; [7]) and the Social Responsiveness Scale (SRS; [8]) (see [9] for a review). These measures show high sensitivity and specificity for detecting formally diagnosed cases of autism and provide a useful tool for identifying people who might benefit from clinical assessment [1, 10]. In addition, typical samples with high levels of autistic traits have a socio-cognitive profile similar to that of people with autism, including greater emphasis on local information during perceptual processing [11–13], more consistent decision-making [14], impaired face-recognition [15], and reduced prosociality [16].

Autistic traits also correlate with socio-economic outcomes. For example, university students studying science subjects have higher AQ scores than students taking arts and humanities subjects, and within the sciences, students of mathematics score higher than those taking physical science or engineering, who in turn score higher than biologists and medics [1] (see also [17]). AQ scores are also higher in those who work in science, technology, engineering, and mathematics (STEM) [10]. Other studies have found a negative relationship between autistic traits and school performance [18, 19]. High AQ scores also predict lower relationship satisfaction [20] and greater loneliness [21] (here and throughout, we use “predict” in the statistical sense of regression analysis, where the dependent variable can be predicted from one or more independent variables, or “predictors”; this does not, of course, establish the causal direction of any relationship between variables, as we discuss below).

This paper investigates the link between autistic traits and another key socio-economic variable: income. In industrialized, market-based economies, income is a key determinant of one’s ability to acquire goods and services. It therefore plays a fundamental role in determining a person’s material circumstances, including their possessions, accommodation, neighbourhood, and access to technology, and predicts a wide variety of social, economic, and health outcomes. Broadly speaking, higher levels of income predict longer life [22], better physical and mental health [23, 24], and greater reported life-satisfaction [25], although the advantages of increasing income may plateau beyond a certain point [25] and there is mounting evidence that how a person’s income compares with a relevant reference group is more important than its absolute value [26]. Income can be difficult to measure, but even broad, categorical reports of household income provide a valid predictor of important outcomes [27–29].

The current paper provides an initial investigation of whether, among typical samples, income is related to a person’s position on the autism spectrum, after controlling for demographic and situational variables. The research comprises five studies: Studies 1 and 2 were not intended as tests of the link between autistic traits and income; rather, income was recorded as a demographic control variable. Because analysis of these preliminary studies found a consistent and unexpected association between autistic traits and income, studies 3–5 were conducted to probe this relationship further.

## Methods

All studies were conducted online using participants recruited via Amazon’s Mechanical Turk (MTurk). MTurk does not perfectly represent the US population, but it is more representative than the student and convenience samples used in many studies [30] and has been used in studies of the cognitive and social correlates of autistic traits [10, 14, 31–34].

Study 1 investigated whether autistic traits predict the tendency to make social comparisons [35]; study 2 investigated whether autistic traits modulate contextual effects on financial decisions [36]. Both studies concluded by requesting demographic information: age, gender, and two simple measures of socio-economic status taken from previously published work: education level, measured on a 4-point scale, and annual pre-tax household income, measured using a 7-point scale [37, 38]. As described below, both studies found an unanticipated negative correlation between income and autistic traits, which led to studies 3–5 being conducted as “purpose-built” explorations of this association.

Whereas studies 1 and 2 used relatively coarse measures of income and education, study 3 elicited a more precise measure of the participant’s household income by using a 16-point scale and adjusted this by the size of the household to obtain a better measure of affluence [26]. Study 4 fractionated household income by asking participants separate questions about their personal income and the income of the rest of their household. Both studies also employed a fine-grained assessment of educational attainment and employment status, and both also examined participants’ subjective socioeconomic status (SSS)—their sense of how they stand relative to the rest of the US population. SSS is positively related to income, but is distinct: an affluent person can believe herself to be relatively poorly off, and vice-versa [39], and these subjective assessments predict many social and psychological outcomes over and above the effects of objective income or wealth [40, 41]. Finally, study 5 examined whether the association between autistic traits and income arises because people with high AQ scores are less likely to exaggerate their wealth. Wealth is often regarded as socially desirable, but people with autism may be less concerned with social reputation [42, 43] and therefore less inclined to overstate their income. Study 5 therefore tested whether the negative relationship between AQ and income persists after controlling for the tendency to make socially desirable responses; it also controlled for the effects of ethnicity.

Ethical approval for all five studies was granted by the University of Cambridge Psychology Research Ethics Committee (PRE.2015.070). Informed consent was obtained from all participants included in the studies. The datasets generated and analysed during the current study are available in the University of Cambridge data repository (https://doi.org/10.17863/CAM.13942).

### Measures

*Autistic traits* were measured with the AQ-Short [44] (hereafter AQ), which consists of 28 items (item order was randomized in all studies). The total score across the 28 items gives an overall assessment of the level of autistic traits. Studies 1, 2, and 5 followed Hoekstra et al. [44] and used a 4-point scale (definitely agree, slightly agree, slightly disagree, definitely disagree). Studies 3 and 4 used a 6-point scale (from “definitely disagree” to “definitely agree”); the labels for the intermediate points on this response scale did not display properly, but the consistency scores were near identical to those reported by Hoekstra et al. [44], indicating that this was not a problem. In studies 3–5, an additional attention check item was included (“Attention check: Please select strongly disagree for this item”) to eliminate unengaged participants. Participants in study 1 also completed the SATQ, comprising 24 items answered on a 4-point scale (not at all true, slightly true, mainly true, very true, coded 1–4 with 15 items reverse-scored).

#### Income

All studies measured household income using an approach found in many previous studies. In studies 1 and 2, participants were asked “What is your annual household income (before taxes)?” with response options: less than $15,000; $15,001 to $25,000; $25,001 to $35,000; $35,001 to $50,000; $50,001 to $75,000; $75,001 to $100,000; $100,001 to $150,000; and greater than $150,000 (e.g. [37]).

Study 3 used a more refined 16-point scale ranging from “less than $10,000” to “more than $150,000” in steps of $10,000, with the option to select “This question is not applicable to me”. This was followed by questions asking how many adults and how many children are in the household. Studies 4 and 5 gave a definition of “household” and asked for the number of adults and children in the household, followed by a question about the participant’s personal pre-tax annual income. If there were other people in the household, the participant was then asked to report the total income of all other members of the household. Both questions used the same 16-point scale as study 3, and both gave the option to indicate that the participant or other householders did not have an income.

Income responses were coded using the mid-point of each category with a median-based Pareto-curve estimator used for the unbounded top category [45, 46]; responses indicating no income for a person or household were coded as $0. For studies 4 and 5, net household income was computed as the sum of own income and other householders’ income.

#### Education

Studies 1 and 2 asked “What is your highest level of educational attainment?” with four response options: did not finish high school, high school graduation, college graduation, and postgraduate degree, coded 1–4. Studies 3–5 asked “What is the highest degree or level of school you have completed? If currently enrolled, mark the previous grade or highest degree received”, with responses on a 12-point scale ranging from “No schooling completed” to “Doctorate degree (for example: PhD, EdD)” coded 1–12.

#### Employment

Participants in studies 3–5 were asked “Which of the following best describes your current situation?” with response options: employed for wages, self-employed, out of work and looking for work, out of work but not currently looking for work, a homemaker, a student, retired, unable to work, other.

#### Subjective socioeconomic status (SSS)

Participants in studies 3–5 completed a widely used measure of subjective status in which they were shown a cartoon ladder representing “where people stand in the United States”, with “the best off – those who have the most money, the most education and the most respected jobs” at the top and “the worst off – who have the least money, least education, and the east respected jobs or no job” at the bottom. Participants selected one of the 10 rungs to indicate where they stand relative to other people in the USA at this time [47].

#### Social Desirability Scale (SDS)

Participants in study 5 completed Stober’s Social Desirability Scale (SDS-17; [48]) by answering True (coded 1) or False (coded 0) to 17 statements such as “I sometimes litter” (6 items are reverse-scored). Higher scores indicate a stronger tendency to make socially desirable responses.

#### Social comparison tendency

Study 1 included the short-form Iowa-Netherlands Comparison Orientation Measure (INCOM; [35]) with responses on a 6-point scale from strongly disagree to strongly agree (coded 1–6; one item reverse-scored). Full details are in the Additional file 1.

#### Financial judgment task

Participants in study 2 completed a simple task, adapted from previous studies [49], in which they were told that they had $80 to invest and rated the attractiveness of different savings accounts. Full details are given in Additional file 1.

#### Demographics

Participants in all studies indicated their gender (male, female; studies 3–5 added the option “prefer not to say”), age (using a slider from 0 to 100), and whether they had ever been diagnosed with autism spectrum disorder or one of its constituents (autism, Asperger’s syndrome, or PPD-NOS; response options: yes, no, prefer not to say). In study 5, participants selected indicated their ethnicity by selecting from eight categories; participants who indicated more than one category were coded as “mixed ethnicity”.

### Design and procedure

In study 1, participants completed the SATQ, INCOM, and AQ, in random order, then indicated their household income, education, and demographics. In study 2, participants completed the financial judgment task followed by the AQ, then household income, education, and demographics. Participants in study 3 completed the AQ followed by the education, employment, and income questions in random order, and finally the SSS followed by the demographic questions. In study 4, participants completed the AQ, education, employment, income, and SSS questions in random order, followed by demographics. Study 5 was intended to be identical to study 4 but with the addition of the Social Desirability Scale and the inclusion of ethnicity in the demographics section. Unfortunately, the survey software malfunctioned: the intention was that participants would complete the AQ, SDS, education, employment, income, and SSS measures in random order. However, one of these items was randomly dropped for each participant. (All participants completed the demographic questions, which appeared at the end of the survey.) Because AQ, income, and SDS were the primary variables of interest, only participants who completed these three items were included in the analyses. Because none of these participants had data for all of the education, employment, and SSS measures, these variables were excluded from the analyses.

### Participants

Eligible participants were respondents who completed the task, who indicated their age as over 18, and whose ID/ip address had not occurred earlier in series of studies (see, e.g. [50]). Participants were excluded prior to data analysis for failing the attention check (*N*_Study_1_ = 17; *N*_Study_2_ = 12; *N*_Study_3_ = 22; *N*_Study_4_ = 43; *N*_Study_5_ = 25), answering “yes” or “prefer not to say” when asked if they had an ASC (*N*_Study_1_ = 4; *N*_Study_2_ = 4; *N*_Study_3_ = 7; *N*_Study_4_ = 23; *N*_Study_5_ = 9), for reporting confusion about the AQ response scale (*N*_Study_3_ = 1; *N*_Study_4_ = 1) or for indicating a household, including themselves, of size zero (*N*_Study_2_ = 2). The final sample sizes and participant information are presented in Table 1. The commonest employment status was “employed for wages” (study 3: 66.75%; study 4, 63.02%, followed by “self-employed” (study 3: 14.25%; study 4: 13.48%). In studies 1 and 2, the modal level of education was “college graduate” (study 1 = 46.99%; study 2 = 56.00%); in studies 3 and 4, it was “bachelor’s degree” (study 3 = 39.75%; study 4 = 40.76%). In study 5, 77% of participants reported being white. Full details about employment, education, and ethnicity are reported in Additional file 1.

**Table 1:** Participant information

	Study 1	Study 2	Study 3	Study 4	Study 5
N	183	350	400	979	579
Male	64.5%	60.9%	60.5%	51.4%	46.5%
Gender not given	–	–	0.5%	0.2%	0.9%
Age range and mean	18–64 32.53 (9.91)	19–75 36.52 (11.12)	20–75 37.34 (12.17)	18–79 37.43 (12.09)	19–84 36.80 (11.91)
Net household income	$36,709 ($2256)	$41,273 ($2139)	$40,257 ($2392)	$60,744 ($2337)	$59,689 ($2335)
Adjusted household income	–	–	$21,666 ($2260)	$29,018 ($2051)	$27,827 (2125)
Personal income	–	–	–	$26,641 ($2919)	$24,798 ($2932)
Others’ income	–	–	–	$32,939 ($3785)	$35,950 ($3268)
SSS	–	–	4.69 (1.65)	4.91 (1.76)	–
AQ	65.18 (11.73)	66.23 (11.16)	92.15 (16.04)	93.82 (15.22)	66.92 (9.83)
% live alone	–	–	29.75%	22.98%	21.93%
Adults	–	–	1.97 (0.91)	2.15 (1.01)	2.16 (1.04)
Children	–	–	0.42 (0.91)	0.50 (0.92)	0.63 (1.04)

Values in parentheses are standard deviations. AQ refers to scores on the short-form Autism Spectrum Quotient. For income measures, the values are geometric means calculated by exponentiating the arithmetic mean of ln(*x* + 1) where *x* is the income in thousands per year; similarly, the income standard deviations are the exponentiated standard deviation of ln(*x* + 1). Others’ income is the net income of all other members of the household, for those participants who do not live alone (*N* = 754 and *N* = 452 for studies 4 and 5, respectively). Studies 3 and 4 used a 6-point response scale for the AQ, which is why the means are much higher than for other studies. % live alone indicates the proportion of participants whose household consists of just one person; adults and children are the mean number of adults (including the participant) and children in the household

### Data analysis

The data provide several measures of income. *Net household income* is the total reported pre-tax income for the household. To obtain a better measure of spending power, for studies 3–5 net income was divided by (number of adults + 0.5 × number of children) (e.g. [26]) to give *adjusted household income* (the unadjusted net income values are also reported, for comparison with the studies 1 and 2). Studies 4 and 5 provide separate measures of the participant’s own income (*personal income*) and of the other people in their household, if any (*others’ income*). All four of these income measures were log transformed (as ln(*x* + 1)) to improve normality (e.g. [26, 46]).

The data were analysed with Pearson’s correlations and multiple linear regressions. In the regression analyses, continuous predictors were standardized and categorical predictors were weighted-effect coded, such that each coefficient tests the effects of category membership against the sample mean (e.g. whether being employed for wages raises or lowers income relative to the overall mean income) [51]. All confidence intervals are 95% confidence intervals.

## Results

### Studies 1 and 2

In study 1, the two measures of autistic traits (AQ scores and SATQ scores) were strongly correlated, *r* = .825, CI = [0.772, 0.866], *p* < .001. Both AQ and SATQ scores were negatively correlated with INCOM-Opinion, indicating that people with higher levels of autistic traits are less concerned about the beliefs of others: *r*_AQ_ = − .193, CI = [− 0.329, − 0.049], *p* = .009; *r*_SATQ_ = − .189, CI = [− 0.325, − 0.045], *p* = .011. In contrast, autistic traits were not meaningfully associated with INCOM-Ability, *r*_AQ_ = − .040, CI = [− 0.184, 0.106], *p* = .592; *r*_SATQ_ = .050, CI = [− 0.096, − 0.193], *p* = .505, indicating little relation between autistic traits and comparison with other people’s abilities. In study 2, there was no effect of AQ on the financial judgment task (Additional file 1).

More importantly for the present paper, both studies found an unexpected negative correlation between AQ and net household income (Table 2). This association remained robust after controlling for age, gender, and education (Table 3). In study 1, the same pattern was found for SATQ scores: SATQ negatively correlated with net household income, *r* = − .232, CI = [− 0.364, − 0.90], *p* = .002, and this effect held when SATQ, age, gender, and education were entered as simultaneous predictors in a regression analysis, *B*_SATQ_ = − 0.176, CI = [− 0.292, − 0.060], *p* = .003.

**Table 2:** Correlations between AQ and other variables

Variable	Study	*r*	95% CI	*p*
Net household income	1	− .250	− 0.381, − 0.109	.001
	2	− .152	− 0.253, − 0.048	.004
	3	− .120	− 0.216, − 0.022	.016
	4	− .204	− 0.263, − 0.143	< .001
	5	− .125	− 0.204, − 0.044	.003
Adjusted household income	3	− .130	− 0.225, − 0.032	.009
	4	− .194	− 0.254, − 0.133	< .001
	5	− .121	− 0.200, − 0.039	.004
Personal income	4	− .156	− 0.217, − 0.095	< .001
	5	− .153	− 0.232, − 0.073	< .001
Others’ income	4	− .096	− 0.166, − 0.025	.008
	5	− .051	− 0.143, 0.041	.279
SSS	3	− .171	− 0.265, − 0.074	.001
	4	− .243	− 0.301, − 0.183	< .001
Age	1	.009	− 0.137, 0.153	.908
	2	− .064	− 0.168, 0.041	.229
	3	.039	− 0.059, 0.137	.433
	4	− .079	− 0.141, − 0.016	.014
	5	− .070	− 0.151, 0.011	.092
Gender	1	.116	− 0.030, 0.257	.118
	2	.040	− 0.065, 0.144	.460
	3	− .026	− 0.124, 0.073	.607
	4	.081	0.018, 0.143	.012
	5	− .022	− 0.104, 0.060	.599
Education	1	− .126	− 0.266, 0.020	.090
	2	.030	− 0.075, 0.134	.578
	3	.009	− 0.090, 0.107	.865
	4	− .094	− 0.156, − 0.031	.003
	5	.027	− 0.071, 0.125	.587

Others’ income refers to the net income of all other members of the household, calculated for those participants who live with other people (*N* = 754 and *N* = 452 for studies 4 and 5, respectively)

*SSS* subjective socioeconomic status

**Table 3:** Regression coefficients for AQ predictor

Dependent variable	Study	Control variables	*B*_AQ_	95% CI	*p*	*R* ^2^_adj_	*R* ^2^_change_
Net household income	1	Age, gender, education	− 0.186	[− 0.300, − 0.071]	.002	.099	.050
	2	Age, gender, education	− 0.121	[− 0.197, − 0.044]	.002	.095	.025
	3	Age, gender, education, employment	− 0.068	[− 0.149, 0.014]	.103	.150	.006
	4	Age, gender, education, employment	− 0.139	[− 0.189, − 0.089]	< .001	.148	.026
	5	Age, gender, ethnicity, socially desirable responding	− 0.115	[− 0.185, − 0.044]	.001	.022	.017
Adjusted household income	3	Age, gender, education, employment	− 0.058	[− 0.132, 0.016]	.127	.197	.005
	4	Age, gender, education, employment	− 0.098	[− 0.138, − 0.058]	< .001	.254	.018
	5	Age, gender, ethnicity, socially desirable responding	− 0.092	[− 0.154, − 0.030]	.004	.049	.014
Personal income	4	Age, gender, education, employment	− 0.105	[− 0.159, − 0.052]	< .001	.395	.009
	5	Age, gender, ethnicity, socially desirable responding	− 0.141	[− 0.228, − 0.055]	.001	.084	.016
Others’ income	4	Age, gender, education, employment	− 0.120	[− 0.215, − 0.024]	.015	.042	.008
	5	Age, gender, ethnicity, socially desirable responding	− 0.086	[− 0.200, 0.027]	.137	.010	.005
SSS	3	Age, gender, education, employment, adjusted household income	− 0.191	[− 0.334, − 0.048]	.009	.275	.013
	4	Age, gender, education, employment, adjusted household income	− 0.222	[− 0.312, − 0.132]	< .001	.376	.015

All regression entered AQ and the control variables simultaneously. Continuous predictors were standardized

*SSS* subjective socioeconomic status, *R* ^*2*^_*adj*_ adjusted *R*-squared for the regression model, *R* ^*2*^_*change*_ difference in (unadjusted) *R* ^2^ between models that do/do not include AQ as a predictor (i.e. the proportion of variance accounted for by AQ, after controlling for other variables)

These studies provided preliminary evidence that autistic traits among the non-clinical population negatively predict income. Because these studies were not intended as test of this relationship, the results must be treated as exploratory. All subsequent results refer to studies 3–5, which used better measures of income and were specifically intended to test the association between income and AQ.

### Household income

In studies 3–5, net and adjusted household income were negatively correlated with AQ (Table 2). To see whether AQ predicted household income over and above the effects of other demographic variables, household income was regressed on AQ, age, gender, education, and employment status. (This analysis was only applied to studies 3 and 4 because study 5 did not have complete education and employment data for all participants.) The coefficients for the AQ predictor are shown in Table 3: for both studies, net income and adjusted income were negatively related to AQ, but the effects were only significant in study 4. To test whether this represents a meaningful difference between the studies, the data from studies 3 and 4 were combined in a regression analysis that included the original variables as well as study and its interactions with all other predictors. Study was weighted-effect coded (with study 3 coded −1), and interactions were computed as described in [52]. With adjusted household income as the dependent variable, the study coefficient was positive, *B*_Study_ = 0.086, CI = [0.064, 0.108], *p* < .001, indicating that incomes were higher than average in study 4. However, none of the interactions involving study were significant (all *ps* > .250) suggesting that the effects of the predictors were consistent across studies. In particular, income was negatively related to AQ, *B*_AQ_ = − 0.086, CI = [− 0.121, − 0.051], *p* < .001, and this effect was not modulated by study, *B*_Study.AQ_ = − 0.014, CI = − 0.038, 0.010], *p* = .260. The same pattern was found when net household income was the dependent variable (*B*_Study_ = 0.118, CI = [0.091, 0.145], *p* < .001; *B*_AQ_ = − 0.118, CI = [− 0.160, − 0.075], *p* < .001; *B*_Study.AQ_ = − 0.024, CI = [− 0.053, 0.005], *p* = .108). Thus, there is a consistent negative association between both income measures and AQ scores, after controlling for other demographic variables.

### Socially desirable responding

The tendency to give socially desirable responses, which was measured in study 5, was negatively related to AQ, *r* = − .179, CI = [− 0.257, − 0.099], *p* < .001. However, the negative relationship between AQ and household income remained after controlling for age, gender, ethnicity, and socially desirable responding (Table 3), suggesting that the association is not purely due to greater honesty from people with high AQ scores (that is, the association between AQ and income is not simply due to people with high levels of autistic traits being less likely to over-state their income in order to inflate their social status).

### Deconstructing household income

In studies 4 and 5, participants separately reported their personal income and, where applicable, the income of the rest of their household (others’ income). In both studies, personal income was negatively related to AQ (Table 2); this effect remained after controlling for age, gender, education, and employment (study 4) and after controlling for age, gender, ethnicity, and socially desirable responding (study 5) (Table 3).

AQ also negatively correlated with others’ income, but the relationship was only significant in study 4 (Table 2); the same pattern was found after controlling for other variables (Table 3). (Cross-study comparison is not possible here because the studies had different predictors.) Notably, the overall variation explained by the predictors is markedly less than for the participant’s own income, as would be expected given that the predictors—including AQ— are likely to have much more relevance to a person’s own economic performance than to the incomes other people in their household.

### Subjective socioeconomic status

SSS was also negatively correlated with AQ (Table 2), and the association remained when SSS was regressed onto AQ, age, gender, education, employment, and adjusted household income (Table 3). That is, after controlling for demographic variables and objective determinants of socio-economic status, people with higher levels of autistic traits put themselves lower on the “socioeconomic ladder” than did people with low AQ scores.

### Subscales

The AQ is intended to capture five distinct types of trait: social skills, preference for routine, task-switching, imagination, and an interest in numbers and patterns. The first four of these constitute a higher-order “social behaviour” factor. To explore the contributions of these different components of the autistic profile, scores for the social behaviour and numbers-and-patterns dimensions were computed by summing the responses to the relevant items [44] and used as the independent variables in a series of regression analyses, one for each of the key dependent variables: adjusted household income, personal income, and subjective socioeconomic status. The regression results are shown in Table 4. The social behaviour dimension was a consistent negative predictor of household income, personal income, and subjective socioeconomic status across all three studies. In contrast, the numbers-and-patterns dimension showed little relationship with household income and was positively related to personal income and subjective socioeconomic status.

**Table 4:** Regression coefficients for social behaviour and numbers-and-patterns sub-traits

Dependent variable		Social behaviour			Patterns-and-numbers			*R* ^2^_adj_
	Study	*B*	95% CI	*p*	B	95% CI	*p*	
Adjusted household income	3	− 0.120	− 0.200, − 0.040	.003	0.030	− 0.050, 0.110	.462	.019
	4	− 0.153	− 0.197, − 0.109	< .001	0.023	− 0.021, 0.068	.301	.046
	5	− 0.096	− 0.158, − 0.035	.002	− 0.002	− 0.063, 0.060	.955	.013
Personal income	4	− 0.194	− 0.260, − 0.128	< .001	0.074	0.008, 0.140	.029	.039
	5	− 0.191	− 0.278, − 0.105	< .001	0.052	− 0.035, 0.138	.243	.032
SSS	3	− 0.328	− 0.488, − 0.168	< .001	0.113	− 0.046, 0.273	.164	.042
	4	− 0.481	− 0.588, − 0.374	< .001	0.120	0.013, 0.226	.028	.082

Predictors were standardized and entered simultaneously

*SSS* subjective socioeconomic status, *R* ^*2*^_*adj*_ adjusted R-squared for the regression model

### Robustness checks

The regression results were unchanged when education was treated as a factor rather than a continuous variable, and when Box-Cox transformations were used in place of logarithmic transformations of income, except that in the latter case the weak relation between AQ and others’ income in study 5 became significant, *B* = − 0.591, CI = [1.139, − 0.043], *p* = .034. Similarly, repeating the analyses using binary coding of responses to the AQ items [1] made little difference to the effects of AQ, except that, in study 3, the negative association between AQ and household income became significant (for net household income, *B* = − 0.082, CI = [− 0.164, − 0.001], *p* = .047; for adjusted household income, *B* = − 0.077, CI = [− 0.151, − 0.003], *p* = .041) and, in study 4, the positive effect of the numbers-and-patterns subscale on adjusted household income became significant, *B* = 0.045, CI = [0.000, 0.089], *p* = .048.

We also examined whether the effects of AQ on income and SSS are explained by a higher propensity for people with high AQ to live in single-member households. In studies 3 and 5, people living alone had similar AQ scores to those living with others: study 3, *M*_alone_ = 91.8 (SD = 15.1), *M*_with_others_ = 92.3 (SD = 16.4), *t*(398) = 0.26, *p* = .795; study 5, *M*_alone_ = 66.8 (SD = 10.2), *M*_with_others_ = 67.0 (SD = 9.8), *t*(577) = 0.16, *p* = .876, although there was evidence for elevated AQ scores among people living alone in study 4, *M*_alone_ = 96.1 (SD = 15.2), *M*_with_others_ = 93.1 (SD = 15.2), *t*(977) = 2.54, *p* = .011. We re-ran the regression analyses for net and adjusted household income, own income, and SSS, with living alone included as a categorical predictor; including this variable made little difference to the coefficients for AQ shown in Table 3, and the pattern of significance was identical.

### Excluding participants with the highest AQ scores

To see whether the effects of autistic traits were driven by the participants with the highest AQ scores (who might represent undiagnosed cases of autism), the regression analyses were re-run after excluding participants with AQ scores more than 1.5 SD above the mean of the sample for the relevant study. (Recall that participants who reported a diagnosis of an ASC, or who preferred not to indicate this, were excluded from the samples prior to analysis, so the calculation of mean and SD were based on the scores of participants who did not have an ASC diagnosis. Participant exclusion occurred after standardizing/weighted-effect coding of predictor variables.) The only changes to the pattern of significance in Tables 3 and 4 were that, in study 3, the negative association between AQ and SSS became non-significant, *B* = − 0.094, CI = [− 0.262, 0.074], *p* = .272, as did the negative association between the social behaviour subscale and adjusted household income, *B* = − 0.078, CI = [− 0.159, 0.003], *p* = .061, and in study 4, AQ was no longer a significant predictor of others’ income, *B* = − 0.055, CI = [− 0.168, 0.057], *p* = .336. The pattern of effects for net household income, adjusted household income, and personal income was unchanged. Thus, the negative association between AQ and income is not entirely driven by the participants with the highest AQ scores.

## Discussion

Income is a complex, multiply-determined variable whose relation to any biological or socio-cognitive predictor cannot be definitively established in a single paper. Nonetheless, the present studies provide an important first step in investigating a potentially crucial functional consequence of non-clinical autistic traits. The studies found a robust negative association between autistic traits and income among people with no reported diagnosis of an autism spectrum condition. We focused on household income because this is a widely used index of a person’s financial well-being that predicts many important outcomes. When household income was dissected in studies 4 and 5, the association with autistic traits was primarily due to the participant’s own income, with some indication that AQ also predicts the probability of living with other highly paid individuals. The link between AQ and income remained after controlling for employment status, as well as age, gender, and education (studies 3 and 4) and ethnicity and socially desirable responding (study 5).

These effects are not large in absolute terms, which is unsurprising given how many variables affect financial success [53]. For example, one meta-analysis found that the correlation between salary and gender was only .18 and that for ethnicity was .11 (3.2 and 1.2% of variance explained, respectively); even the best predictor, education, only correlated at .29 (8.4% variance explained) [54]. Against this background, and given the importance of income to well-being, the correlations and *R* ^2^_change_ values in Tables 2 and 3 suggest that AQ has a modest but meaningful relationship with income.

The generality of these findings is an open question. The online samples used here are more similar to the general population than many of the samples used in studies of autistic traits, but they are not perfectly representative. In addition, there is evidence that autistic traits—and other psychopathological traits—are more pronounced in participants recruited through MTurk than in other samples [55]. The mean AQ-Short scores in the current studies are higher than those in some other studies [44], although they are similar to results from Nishiyama et al. [9] who used a diverse sample of students and employees. Very few studies of autistic traits accurately represent a particular national population, and it will obviously be important to test whether the pattern found here generalizes to other samples. Likewise, it will be instructive to use more fine-grained measures of financial circumstances, such as those that correct for regional costs of living [26]. Nonetheless, the population sampled comprises people from widely varying backgrounds and with diverse personal attributes and circumstances [30, 56–58], and the present results generate several important directions and questions.

The first concerns the causal status of the relationship between income and AQ. One possibility is that AQ and income are both caused by an unmeasured confounding variable, such as the presence of other psychiatric conditions: although we excluded participants with a diagnosis of autism, AQ is elevated in conditions including schizophrenia, social anxiety disorder, and obsessive compulsive disorder [59, 60], all of which might negatively affect income. Another is that the social and cognitive consequence of lower income shape a person’s social attitudes and cognitive style in a way that elevates their AQ score. For example, reduced income may lead to reduced opportunity for social interaction, less opportunity to develop imaginative pursuits, poorer nutrition (which could impair task switching), and a more chaotic lifestyle (such that one values routine). All of these would boost a person’s self-reported scores on subdomains of the AQ. However, given that AQ is argued to measure stable socio-cognitive dispositions with a heritable basis [1, 61], the most likely causal direction is for autistic traits to influence a person’s financial circumstances.

This might arise because even subclinical levels of the social atypicalities that characterize autism limit a person’s career progression. Some jobs—such as selling for commission—directly link income to interpersonal interaction and social skills; more generally, “social capital” is an important component of career progression in many fields [62]. In keeping with this, the analysis of subscales suggested that the negative association between AQ and income is driven by atypical social behaviour; in contrast, personal income was positively correlated with the “numbers and patterns” component of the AQ. This accords with evidence that autistic traits are more pronounced among people working in fields such as mathematics and engineering [10], which often offer very high remuneration. Autistic traits may therefore represent something of a double-edged sword.

Autistic traits could also influence income via their effects on economic decision-making. Decision-making is an under-researched aspect of autism [63], but there is emerging evidence that people with autism have a different decision-making style from the general population [64, 65]. For example, Farmer et al. [14] found that people with autism, and non-clinical samples with high levels of autistic traits, show a more conventionally “rational” decision-making style which may not be adaptive in real environments. This atypical profile could limit a person’s financial success. Alternatively, people with high levels of autistic traits may simply have priorities and preferences that do not lead to material wealth.

Establishing the causal relationship between autistic traits and income will require longitudinal studies in which a cohort is followed from early in life, to determine whether and how subclinical autistic traits correlate with subsequent financial outcomes after controlling for other variables, and to see whether changes in income (for example, in response to a pay-cut) lead to changes in AQ score. However, whatever the basis for the negative association between AQ and income, it has important implications because low incomes predict poorer mental and physical well-being and lower life satisfaction [22, 24]. The AQ-income association might therefore partially explain why people with high levels of non-clinical autistic traits are more likely to report socio-psychological problems [18, 66], although it could also be that the problems associated with high levels of autistic traits limit people’s ability to gain control of financial resources. As a broader point, the current data suggest that whenever a researcher finds a link between income and some socio-psychological variable such as materialism [67], interpersonal hostility [38], or prosociality [68], the effects may be due to the greater prevalence of autistic traits among the low-income participants.

A final observation is that AQ was negatively associated with subjective socioeconomic status, even after controlling for objective socioeconomic indicators (education, employment status, and income). This is concerning because negative health, social, and psychological outcomes are often better predicted by a person’s beliefs about their income and social class than by their objective circumstances [26]. However, subjective socioeconomic status per se is less important than the extent to which a person resents his or her relative position [37], so it will be important to explore the link between these reactions and autistic traits.

## Conclusions

These studies provide a first exploration of the link between autistic traits and socio-economic status, and it would be premature to draw firm conclusions at this point. Nonetheless, the negative correlation is sufficiently robust and potentially important that exploring the association will be an important direction for researchers interested in the causes and consequences of subclinical autistic traits.

## Additional file

Additional file 1: This file gives further information about the financial decision task in study 2. It also tabulates the educational background of participants in studies 1–4, employment status for participants in studies 3 and 4, and ethnicities of participants in study 5. (PDF 286 kb)

## Abbreviations

AQ: Autism Spectrum Quotient
INCOM: Iowa-Netherlands Comparison Orientation Measure
MTurk: Amazon’s Mechanical Turk
SATQ: Subthreshold Autism Trait Questionnaire
SDS: Social Desirability Scale
SSS: Subjective socioeconomic status
STEM: Science, technology, engineering, and mathematics
