# Supplementary material for: Initial evidence that non-clinical autistic traits are associated with lower income
Source: Mol Autism. 2017 Nov 13;8:61. doi: 10.1186/s13229-017-0179-z (PMC5683395; doi:10.1186/s13229-017-0179-z)
Supplement: Additional file 1: — This file gives further information about the financial decision task in study 2. It also tabulates the educational background of participants in studies 1–4, employment status for participants in studies 3 and 4, and ethnicities of participants in study 5. (PDF 286 kb) [file 13229_2017_179_MOESM1_ESM.pdf]

SUPPLEMENTARY MATERIALS

Further information about the financial decision task in Study 2

People with autism spectrum conditions show reduced sensitivity to global context identified in many tasks, including some decision-making tasks. Study Supp2 was motivated by the idea that autistic traits in the non-clinical population might modulate the extent to which people's financial judgments are context-dependent.

Method

Materials, Design, and Procedure

Participants completed a simple task modelled on one used in previous studies of context-sensitivity (e.g., [1]) in which they were told that they had $80 to invest and rated the attractiveness of different savings accounts. All of the accounts offered a return of $120, but they matured at different times. In the Unimodal condition, the accounts matured in 4,5,6,7,8,9,10, and 11 months; in the Bimodal condition, they matured in 1, 2, 3, 4, 11, 12, 13, or 14 months. Participants were randomly assigned to one of these two conditions and rated the attractiveness of the accounts on a 10-point scale (1= Very unattractive, 10 = Very attractive). After the judgment task, participants completed the AQ-short followed by the same demographic questions as in Study S1.

Participants

Twelve participants were excluded because they indicated that the shortest delay was less attractive than the longest, indicating a failure to attend to the task; a further four were excluded for answering "yes" (N=2) or "prefer not to say" (N=2) when asked if they had ever been diagnosed with an ASC, leaving a final sample of 350, as described in the main text; 175 were in the unimodal condition and 175 were in the bimodal condition. Education composition was: 0.29% did not finish high school; 34.28% high school graduation; 56.00% college graduation; 9.43% postgraduate degree.

Results

The key test of context sensitivity is the difference in the judged attractiveness of the two times that are common to both conditions: 4 months vs 11 months. According to conventional rank-based accounts of contextual judgment, the difference between 4 months and 11 months will be greater in the Unimodal condition than in the Bimodal condition, because there are more intervening delays (e.g., [2, 3]) .

To test the focal hypothesis, the difference between the attractiveness of the 4-month and 11-month options was regressed on condition (coded: unimodal = -1, bimodal = +1), standardized AQ, and their product. The condition coefficient was negative, B = -0.977, CI = [-1.238, -0.717], p <.001, indicating that people judged the difference between 4 months and 11 months to be larger when there were more intervening durations, as expected. There was no main effect of AQ score, B = 0.009, CI = [-0.252, 0.271], p = .943, and no indication that AQ moderated the effect of condition, B = 0.184, CI = [-0.078, 0.445], p = .168. This pattern was unchanged when age, gender, and household income were included as predictors.

Of more relevance to the current work, Study Supp2 again found a negative correlation between household income and AQ score, and this association remained when income was regressed onto AQ, age, gender, and education like for Study Supp1. These results are shown in the main text.

Table S1: Educational background in Studies 1 and 2

	Study 1	Study 2
Did not finish high school	1.09%	0.29%
High school graduate	45.9%	34.28%
College graduate	46.99%	56.00%
Postgraduate degree	6.01%	9.43%

Table S2: Employment status and Educational background of participants in Studies 3 and 4

	Study 3	Study 4
Employed for wages	66.75%	63.02%
Self-employed	14.25%	13.48%
Out of work and looking for work	6.50%	4.60%
Out of work but not currently looking for work	1.00%	1.12%
A homemaker	3.25%	4.90%
A student	3.25%	6.84%
Retired	4.00%	3.37%
Unable to work	0.75%	2.25%
Other	0.25%	0.41%
No schooling completed	0.25%	0%
Nursery school to 8th grade	0.25%	0.10%
9th, 10th, or 11th grade	0.25%	0.20%
12th grade, no diploma	0.25%	0.31%
High school graduate	11.00%	7.87%
Some college credit, but less than one year	9.25%	8.38%
1 or more years of college, no degree	15.25%	14.4%
Associate degree (e.g., AA, AS)	9.75%	12.16%
Bachelor's degree (e.g., BA)	39.75%	40.76%
Master's degree (e.g., MA	11.00%	11.44%
Professional degree (e.g., MD)	1.25%	2.25%
Doctorate degree (e.g., PhD)	1.75%	2.15%

Note: Although some participants in Study 5 reported their education and employment status, this information was not recorded for all participants and so is not reported here.

Table S3: Ethnicities of participants in Study 5

Ethnicity	Proportion
White (for example, German, Irish, English, Italian, Polish, French, etc)	77.03%
Hispanic, Latino, or Spanish origin (for example, Mexican or Mexican American, Puerto Rican, Cuban, Salvadoran, Dominican, Colombian, etc)	5.35%
Black or African American (for example, African American, Jamaican, Haitian, Nigerian, Ethiopian, Somalian, etc)	5.35%
Asian (for example, Chinese, Filipino, Asian Indian, Vietnamese, Korean, Japanese, etc)	5.35%
American Indian or Alaska Native (for example, Navajo Nation, Blackfeet Tribe, Mayan, Aztec, Nome Eskimo Community, etc)	0.17%
Middle Eastern or North African (for example, Lebanese, Iranian, Egyptian, Syrian, Moroccan, Algerian, etc)	0.52%
Native Hawaiian or Other Pacific Islander (for example, Native Hawaiian, Samoan, Chamorro, Tongan, Fijian, etc)	0.17%
Some other race, ethnicity, or origin	0.52%
Multiple responses selected	5.53%

References

1. Matthews WJ. How much do incidental values affect the judgment of time? Psychol Sci. 2012;23:1432–4. doi:10.1177/0956797612441609

2. Brown GDA, Gardner J, Oswald AJ, Qian J. Does wage rank affect employees' well-being? Ind Relat. 2008;47:355–89. doi: 10.1111/j.1468-232X.2008.00525.x

3. Brown GDA, Matthews WJ. Decision by sampling and memory distinctiveness: Range effects from rank-based models of judgment and choice. Front Psychol. 2011;2:Article 299. doi: 10.3389/fpsyg.2011.00299
